# Nutrition Screening in the Pediatric Intensive Care Unit: Evaluation of an Electronic Medical Record-Based Tool

**DOI:** 10.3390/nu15214591

**Published:** 2023-10-28

**Authors:** Julia Hilbrands, Mary Beth Feuling, Aniko Szabo, Bi Q. Teng, Nicole Fabus, Melissa Froh, Rebecca Heisler, Olivia Lampone, Amber Smith, Theresa A. Mikhailov, Praveen S. Goday

**Affiliations:** 1 Clinical Nutrition, Children’s Wisconsin, Milwaukee, WI 53226, USA; jhilbrands@childrenswi.org (J.H.); mfeuling@childrenswi.org (M.B.F.); nfabus@childrenswi.org (N.F.);; 2 Division of Biostatistics, Medical College of Wisconsin, Milwaukee, WI 53226, USA; aszabo@mcw.edu (A.S.);; 3 Nutrition Services, University of California San Francisco Health, San Francisco, CA 94143, USA; 4 Pediatric Critical Care, Medical College of Wisconsin, Milwaukee, WI 53226, USA; 5 Pediatric Gastroenterology, Nationwide Children’s Hospital, Columbus, OH 43205, USA

**Keywords:** critical illness, malnutrition, nutrition assessment, nutrition support

## Abstract

Hospitalized, critically ill children are at increased risk of developing malnutrition. While several pediatric nutrition screening tools exist, none have been validated in the pediatric intensive care units (PICU). The Children’s Wisconsin Nutrition Screening Tool (CWNST) is a unique nutrition screening tool that includes the Pediatric Nutrition Screening Tool (PNST) and predictive elements from the electronic medical record and was found to be more sensitive than the PNST in acute care units. The aim of this study was to assess the performance of the tool in detecting possible malnutrition in critically ill children. The data analysis, including the results of the current nutrition screening, diagnosis, and nutrition status was performed on all patients admitted to PICUs at Children’s Wisconsin in 2019. All 250 patients with ≥1 nutrition assessment by a dietitian were included. The screening elements that were predictive of malnutrition included parenteral nutrition, positive PNST, and BMI-for-age/weight-for-length z-score. The current screen had a sensitivity of 0.985, specificity of 0.06, positive predictive value (PPV) of 0.249, and negative predictive value of 0.929 compared to the PNST alone which had a sensitivity of 0.1, specificity of 0.981, PPV of 0.658, and NPV of 0.749. However, of the 250 included patients, 97.2% (243) had a positive nutrition screen. The CWNST can be easily applied through EMRs and predicts the nutrition risk in PICU patients but needs further improvement to improve specificity.

## 1. Introduction

Hospitalized children are at increased risk of developing malnutrition. Malnutrition, including under- and overnutrition, has been shown to lead to longer lengths of stay, increased morbidity and mortality, longer duration of ventilator support, and increased complications such as organ dysfunction and hospital-acquired infections [1,2,3,4]. For example, a recent study by Bechard and colleagues found that underweight and obese patients had a 29% and 18% lower chance of being discharged when compared to well-nourished patients, respectively [5]. Undernutrition can impair the immune system and dampen the body’s ability to fight infections, and it can also reduce the effectiveness of respiratory muscles, leading to respiratory challenges and a greater need for ventilator support [6].

In addition to the risks of undernutrition, critically ill children often have increased nutrient needs in the setting of increased metabolic stress and inflammation, and their nutrition status can deteriorate quickly due to barriers to receiving nutrition such as fluid restriction, intermittent nil per os (NPO) times for procedures and feeding intolerance or malabsorption [1]. Malnutrition prevalence in the pediatric intensive care unit (PICU) at admission ranges widely from 8 to 72% [7,8]. This variance is dependent on a variety of factors including different diagnostic criteria for malnutrition. Given the likelihood of poorer outcomes with malnutrition, the PICU nutrition support guidelines recommend that all patients in the PICU receive a nutrition assessment within 48 h of admission and that nutrition status be reevaluated at least weekly [2]. As such, it is important to have a validated screening method and monitoring tool for the PICU patient population to identify malnutrition and provide early implementation of nutrition interventions.

A variety of screening tools have been created to help clinicians focus resources on the patients with the highest nutrition risk to promote improved patient outcomes, but no nutrition screens have been validated in the PICU population to date [6]. A systematic review completed by Ventura et al. (2022) identified 19 nutrition screens for assessing nutrition status in hospitalized patients; however, only 6 of the studies even reported including PICU patients. Some of these screening tools do show a high sensitivity for identifying nutrition risk in acute care units, but since none of them have been validated in critically ill children, it has been recommended that new screening tools should be developed for this population [6,9,10,11].

The Joint Commission in the USA requires that all hospitalized patients be screened for malnutrition within 24 h of admission. In general, nutrition screens need to be concise since they have to be administered to each patient at admission. The Children’s Wisconsin Nutrition Screening Tool (CWNST) is a unique tool created by the dietitians at the Children’s Wisconsin (CW) hospital that is embedded in the electronic medical record (EMR) and with that advantage, it contains many more elements which can be automatically obtained from the EMR. The CWNST is described below and was previously evaluated to determine which screening elements were associated with malnutrition risk in the acute care population [12]. We hypothesized that the additional data elements would make the tool more accurate than prior screening tools. The aim of the current study was to assess if this tool was able to predict nutrition risk and the association between elements of this screen and malnutrition risk specifically in the PICU population. Since a screening tool can still be valuable if it is able to identify only more severe levels of malnutrition, we also aimed to assess its relationship with any malnutrition as well as with moderate and severe malnutrition alone.

## 2. Materials and Methods

### 2.1. Development of the Children’s Wisconsin Nutrition Screening Tool

The development of the CWNST has been described in detail previously [12]. Briefly, this screen was developed based on the ASPEN Pediatric Nutrition Care Pathway, the Academy of Nutrition and Dietetics (AND)/ASPEN pediatric malnutrition criteria, as well as from the clinical experience of the RDs and medical providers at CW [13,14]. The screen was developed to be a daily patient-centered screening tool that makes use of automated reporting in electronic medical records (EMRs) so that every hospitalized pediatric patient can be efficiently screened every day for nutrition risk.

The screen is a set of elements to which the responses are binary (yes/no) and we aimed to identify the set of questions that, without or without the PNST, would be the most sensitive and specific for nutrition risk. The elements of the CWNST include the PNST along with 7 additional daily screening elements: enteral nutrition (EN), parenteral nutrition (PN), presence of intubation, 2+ food allergies, BMI-for-age z-score (BMIZ) < −1 (children ≥ 2 years), weight-for-length z-score (WFLZ) < −1 (children < 2 years), as well as an item called Registered Dietitian (RD)-identified risk. RD-identified risk is something that RDs can select if their clinical judgment suggests a patient is at nutrition risk even if the other screening criteria are not met (i.e., a high-risk diagnosis or high degree of weight loss) and is a subjective element indicated by the RD. For instance, RD-identified risk might be used to indicate a high-risk diagnosis (such as cystic fibrosis) or for a patient determined to be at-risk based on visual assessment.

The CWNST also contains 3 status change elements that are assessed on day 4 of hospitalization and three times per week thereafter; these status change elements are intake < 50% of needs, NPO status, and unintentional weight loss of ≥5%, each over 3 or more days.

### 2.2. Screening Process

The daily screening process has also been outlined previously [12]. In summary, a nurse administers the PNST upon hospital admission and enters this into the EMR, and then the EMR automatically generates the remaining 7 screen elements every day at 6:00 a.m. If any screen element is positive for a patient, the unit RD can see this in their patient list in the EMR; they review the information in the EMR and apply their clinical judgment to determine if they think the patient should receive a full nutrition assessment with nutrition interventions as needed. This process is repeated every day at 6 am so that if a patient’s medical or nutritional status changes (i.e., the patient is intubated), this will be identified on the screen the following day.

Additionally, on day 4 of hospitalization and every Monday, Wednesday, and Friday thereafter, the unit RD assesses the 3 status change elements. The NPO status is automatically generated by the EMR, but intake < 50% of needs and unintentional weight loss of ≥5% must be reviewed manually.

### 2.3. Data Collection

The evaluation of the CWNST in the PICU population was a retrospective cohort study that included all patients who were admitted to the PICU at CW in 2019 who were ≤17 years old and who were assessed by an RD. Patients from the Neonatal Intensive Care Unit (NICU) were excluded, and data from patients on acute care units have been reported previously [12]. The data obtained included age, length of stay, primary diagnosis (including presence of a childhood chronic condition (CCC), defined as any medical condition that typically lasts ≥12 months and involves one or more organ systems severely enough to require specialty care and possibly hospitalization in a tertiary care center [13]), results of the CWNST (including that of the PNST), and malnutrition assessment. Malnutrition was classified based upon RD assessment according to AND/ASPEN malnutrition indicators [14,15].

CW Institutional Review Board approval (IRB # 1599359) was obtained prior to study commencement.

### 2.4. Statistical Analysis

Descriptive data were used to describe the patient demographics. Individual screening elements were assessed using Fischer’s exact test, and WFLZ and BMIZ were combined into a single variable for analysis as these variables are mutually exclusive (WFLZ is used for patients less than two years old corrected age while BMIZ is used for patients two years old corrected age and greater).

A risk factor analysis was completed to assess which combination of screening elements produced the best predictability for malnutrition. The steps for this analysis have been described previously [12]. Briefly, a weighted prediction quality score was computed by assigning penalty weights of 1, 3, and 5 to failing to predict mild, moderate, or severe malnutrition, respectively, and a penalty weight of 0.2 for predicting malnutrition when there was none. The model with the lowest penalty score was then selected. Of note, since the status change elements occurred with very low frequency, they were not included in this analysis. The risk factor analysis was completed to examine how well the CWNST identified all levels of malnutrition, and then repeated with just moderate and severe malnutrition (wherein patients with no malnutrition and mild malnutrition were grouped together).

We also assessed the functionality of the PNST alone in the study population to serve as a source of comparison with the full CWNST. The sensitivity, specificity, PPV, and NPV of the PNST to predict any degree of malnutrition as well as moderate and severe malnutrition only was calculated and compared to these measures of the CWNST as a whole. The focus was on not missing malnutrition, especially moderate or severe, so an overall sensitivity of 95% (or higher) and 99% or higher among patients with moderate/severe malnutrition was the target. A 5% significance level was used.

The analyses were performed using R version 4.0.3.

## 3. Results

**Figure 1:**
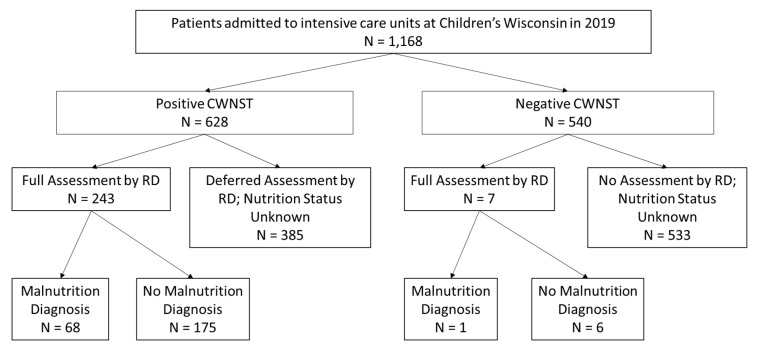
Flowsheet of Patients included in the Study.

Of the 1168 patients whose data were reviewed, the 250 patients who had ≥1 RD assessment were included in the risk factor analysis (Figure 1). The remainder (918 patients) did not undergo an RD assessment and thus their true nutrition status is unknown. The patients who received an RD assessment were younger, had a longer length of stay, and were more likely to have a chronic childhood condition than patients who were not assessed (see Table 1).

### 3.1. Diagnosis of Malnutrition and Nutrition Screen Results

Of the 250 patients with nutrition assessment by an RD, 181 patients (72.4%) had no malnutrition while 69 patients (27.6%) had malnutrition of some severity: 27 patients (10.8%) had mild malnutrition, 24 patients (9.6%) had moderate malnutrition, and 18 patients (7.2%) had severe malnutrition. For these 250 patients, 97.2% (243) had a positive nutrition screen, and the frequency of each positive nutrition screen element and the prevalence of the associated maximum malnutrition diagnosis can be found in Table 2. Significant differences between malnourished and normally nourished children were seen with the following elements: parenteral nutrition (PN), positive PNST, and BMIZ or WFLZ < −1. Of the 918 patients who were not assessed by an RD, 41.9% had a positive CWNST (see Table 3 and Figure 1).

### 3.2. Nutrition Risk Factor Selection

Based on the misclassification penalty score computations, 5–6 screening elements produced the lowest penalty score, and this combination of screening elements was best at efficiently identifying malnutrition (see Table 4). When this analysis was re-run to predict just moderate and severe malnutrition, a 4–5 screening element combination generated the lowest penalty score (see Table 5).

### 3.3. PNST

We also assessed the functionality of the PNST alone in PICU patients. The PNST had a sensitivity of 0.1, specificity of 0.981, PPV of 0.658, and NPV of 0.749 at predicting all levels of malnutrition and had a sensitivity of 0.141, a specificity of 0.975, PPV of 0.474, and NPV of 0.878 at predicting just moderate and severe malnutrition (see Table 6).

## 4. Discussion

The aim of the current study was to assess whether the Children’s Wisconsin Nutrition Screening Tool is able to predict nutrition risk and the association between elements of this screen and malnutrition risk within a population of PICU patients, and the results showed that several screening elements have significant predictive value for a malnutrition diagnosis. PN, positive PNST, and BMIZ or WFLZ < −1 were statistically significant in identifying patients with malnutrition when compared to other elements of the screen. This correlates with the ASPEN Critical Care Guidelines to obtain the weight and height or length of all patients admitted to the PICU in order to calculate a WFLZ or BMIZ to screen for patients at greatest nutrition risk [2]. Interestingly, this is not consistent with the recommendations from Ventura and colleagues to include the degree of severity of illness and presence of chronic disease in a nutrition screen; if so, we might expect to see additional screening elements such as intubation and RD-identified risk to be significant predictors of malnutrition [6].

The CWNST in critically ill children is a highly sensitive screen, and the sensitivity of the screen increased further when it was used to identify just moderate and severe malnutrition. This high degree of sensitivity of the screen has emphasized that almost all PICU patients are at risk of malnutrition during their stay and benefit from being monitored daily by an RD, even if a full assessment and/or nutrition intervention is not required. Interestingly, EN as an individual screening element was not predictive of malnutrition. However, when EN, PNST, and BMIZ or WFLZ < −1 were used together, the predictive value of the CWNST significantly increased, as seen in the risk factor selection analysis.

While the CWNST was highly sensitive, it did include severity of illness as one of its components. It is well established that patients at a high risk of malnutrition have more chronic diseases, and based on a recent systematic review, the severity of illness was one of the key screening variables to identify patients at nutrition risk as this can change over the course of hospitalization in the PICU [6,16]. This trend was observed anecdotally in the study population as patients assessed and followed by an RD had a higher rate of CCC than those who were not.

The Joint Commission requires nutrition screening of all patients at hospitalization but does not specify the screening criteria, although a validated tool is recommended to be used by the ASPEN Pediatric Nutrition Care Pathway for the initial nutrition screen [15]. In our screen, the PNST was used as the validated screening tool along with additional elements recommended by the ASPEN Pediatric Nutrition Care Pathway. However, the PNST was not validated in PICU patients. Of the 69 patients diagnosed with malnutrition in the study population, only 35% had a positive PNST screen, showing some potential limitations of the PNST in a population of PICU patients. When the remaining variables of the CWNST were included, 98.5% of patients diagnosed with malnutrition were identified as at risk.

Only 250 of the 1168 screened patients were assessed by an RD. Of the 918 patients who did not have an RD assessment, 41.9% screened positive but did not require a full assessment by an RD for reasons including but not limited to improving weight for length/BMI, stable enteral nutrition with recent RD assessment that occurred at a different hospital or ambulatory clinic encounter, multiple food allergies that did not impact ability to meet nutritional needs, or intubation of short duration and ability to advance oral intake or ability to resume home enteral nutrition.

The CWNST is highly sensitive and identified the majority of patients at nutrition risk, which is the primary strength of the screen. Another strength of this screen is its design: it is based on a patient list model and embedded almost entirely in EMRs, making it easy and efficient to screen a large number of hospitalized patients on a daily basis and quickly identify those that may require nutrition intervention. This is significant as the nutrition status of critically ill children can change quickly and RD time and availability may be limited in many institutions. It is also an improvement on the recommendations in the ASPEN Critical Care Guidelines to reassess the nutrition status of critically ill patients on at least a weekly basis [2]. As we showed with the PNST, current nutrition screening tools may have significant limitations when used in a PICU population, and the CWNST may be useful in addressing those limitations.

A significant limitation of this study is that 79% of the patients admitted to the PICU did not receive an RD assessment during hospitalization. This could be due to the mean duration of stay <7 days or clinical judgment by the RD that they did not require acute nutrition intervention, but the fact remains that critically ill patients are at greater nutritional risk, and there remains the potential for the development of malnutrition following their PICU admission [1]. Additionally, while the CWNST has a high degree of sensitivity, it is not very specific. Approximately 72.4% of patients who had a positive screen did not receive a malnutrition diagnosis, while 41.9% of patients who did not receive an assessment had a positive screen. This low degree of specificity may limit the screen’s overall efficiency. Removing some of the screen elements—intubation, food allergies, RD-identified risk—leads to some improvement in specificity without a significant impact to sensitivity and could help improve the performance of this screen. With regard to other improvements in the screen, a variety of other questions that have been used in other screens could be easily added but this would likely remove the attractiveness of a mostly automated EMR-based screen. This was a single-center study where race was not taken into account, so it is unknown how generalizable the CWNST is across cultures and ethnicities. Another limitation of this screen and pediatric nutrition screening at large is the lack of any validated nutrition screening tool relevant to the PICU setting. Due to this, we are unable to compare the data obtained in this study against other screening tools or data from other institutions.

## 5. Conclusions

Nutrition screening in the PICU remains a challenge with many patients at nutrition risk in addition to not having a validated screening tool. The CWNST is a unique screening tool that is useful for predicting nutrition risk in PICU patients, can be easily applied through the EMR, and captures more at-risk patients than the PNST alone. However, it needs further improvement to improve specificity and decrease sensitivity in order to provide the most at-risk PICU patients with early nutrition intervention.

## Figures and Tables

**Table 1:** Demographics.

	All	No RD Assessment	RD Assessment	*p*-Value
Total (n)	1168	918	250	
Age (years)				<0.001 ^a^
Mean (SD)	6.06 (5.62)	6.43 (5.65)	4.72 (5.29)	
Median (Min, Max)	3.00 (0.01, 17.00)	4.00 (0.03, 17.00)	2.00 (0.01, 17.00)	
Readmitted during study period	190 (16.3%)	124 (13.5%)	66 (26.4%)	<0.001 ^c^
Length of stay (days)				<0.001 ^a^
Mean (SD)	6.94 (23.95)	2.25 (3.45)	24.18 (47.59)	
Cardiac disorder	60 (5.1%)	44 (4.8%)	16 (6.4%)	0.332 ^c^
Endocrine disorder	15 (1.3%)	13 (1.4%)	2 (0.8%)	0.751 ^c^
Gastrointestinal disorder	25 (2.1%)	13 (1.4%)	12 (4.8%)	0.003 ^c^
Genetic disorder	107 (9.2%)	78 (8.5%)	29 (11.6%)	0.138 ^c^
Neurobehavioral/CNS disorder	106 (9.1%)	100 (10.9%)	6 (2.4%)	<0.001 ^c^
Respiratory disorder	401 (34.3%)	306 (33.3%)	95 (38.0%)	0.177 ^c^
Miscellaneous disorder	471 (40.3%)	375 (40.8%)	96 (38.4%)	0.513 ^c^
Chronic Childhood Condition ^b^	867 (74.2%)	632 (68.8%)	235 (94.0%)	<0.001 ^c^

Abbreviations: SD, standard deviation; CNS, central nervous system. ^a^ Wilcoxon rank-sum test. ^b^ Defined as any medical condition lasting ≥12 months and involves one or more organ system severely enough to require specialty care and possibly hospitalization in a tertiary care center [15]. ^c^ Fisher’s exact test.

**Table 2:** Positive nutrition screens stratified by nutrition status.

Variable	Total (*n* = 250)	No Malnutrition (*n* = 181)	Any Malnutrition (*n* = 69)	*p*-Value *
Any positive screen	243 (97.2%)	175 (96.7%)	68 (98.6%)	0.677
Enteral nutrition	202 (80.8%)	146 (80.7%)	56 (81.2%)	>0.999
>2 food allergies	6 (2.4%)	4 (2.2%)	2 (2.9%)	0.669
Intubation	84 (33.6%)	64 (35.4%)	20 (29.0%)	0.372
Parenteral nutrition	56 (22.4%)	29 (16.0%)	27 (39.1%)	<0.001
RD-identified risk	42 (16.8%)	28 (15.5%)	14 (20.3%)	0.352
Positive PNST	37 (14.8%)	13 (7.2%)	24 (34.8%)	<0.001
BMI-for-age or weight-for-length z-score	72 (28.8%)	32 (17.7%)	40 (58.0%)	<0.001
Intake < 50% for 3 days	10 (4.0%)	7 (3.9%)	3 (4.3%)	>0.999
NPO > 3 days	3 (1.2%)	3 (1.7%)	0 (0.0%)	0.563
Weight loss > 5%	3 (1.2%)	3 (1.7%)	0 (0.0%)	0.563

* Fisher’s exact test. Legend: BMI, body mass index; NPO, nil per os (nothing by mouth); PNST, Pediatric Nutrition Screening Tool; RD, registered dietitian.

**Table 3:** Screen results for patients without a nutrition assessment.

Variable	Total (*n* = 918)
Any positive screen	385 (41.9%)
Enteral nutrition	295 (32.1%)
>2 food allergies	14 (1.5%)
Intubation	102 (11.1%)
Parenteral nutrition	0 (0%)
RD-identified risk	0 (0%)
Positive PNST	27 (2.9%)
BMI-for-age or weight-for-length z-score	121 (13.2%)
Intake < 50% for 3 days	6 (0.7%)
NPO > 3 days	2 (0.2%)
Weight loss > 5%	2 (0.2%)

Legend: RD: registered dietitian; PNST: Pediatric Nutrition Screening Tool; NPO: nil per os.

**Table 4:** Predictability of all levels of malnutrition by screening variables.

N	Score	Screening Variables	Sensitivity	Specificity	PPV	NPV
1	231.6	BMI/WFL z-score	0.309	0.902	0.5	0.805
2	157.2	EN, BMI/WFL z-score	0.853	0.256	0.266	0.846
3	124.4	EN, PN, BMI/WFL z-score	0.941	0.135	0.256	0.879
4	104.8	EN, PN, PNST, BMI/WFL z-score	0.941	0.126	0.254	0.871
5	97.0	EN, PN, RD-identified risk, PNST, BMI/WFL z-score	0.985	0.074	0.252	0.941
6	97.0	EN, PN, food allergies, RD-identified risk, PNST, BMI/WFL z-score	0.985	0.074	0.252	0.941
7	97.6	EN, PN, food allergies, intubation, RD-identified risk, PNST, BMI/WFL z-score	0.985	0.06	0.249	0.929

Legend: BMI, body mass index; EN, enteral nutrition; NPV, negative predictive value; PN, parenteral nutrition; PNST, Pediatric Nutrition Screening Tool; PPV, positive predictive value; RD, registered dietitian; WFL, weight-for-length.

**Table 5:** Predictability of moderate and severe malnutrition using only screening variables.

N	Score	Screening Variables	Sensitivity	Specificity	PPV	NPV
1	195.0	BMI/WFL z-score	0.643	0.877	0.214	0.979
2	149.4	EN, BMI/WFL z-score	1	0.242	0.064	1
3	129.2	EN, PNST, BMI/WFL z-score	0.643	0.658	0.089	0.973
4	111.4	EN, PN, PNST, BMI/WFL z-score	1	0.115	0.056	1
5	107.2	EN, PN, RD-identified risk, PNST, BMI/WFL z-score	1	0.063	0.053	1
6	107.2	EN, PN, food allergies, RD-identified risk, PNST, BMI/WFL z-score	1	0.063	0.053	1
7	109.0	EN, PN, food allergies, intubation, RD-identified risk, PNST, BMI/WFL z-score	1	0.052	0.052	1

Legend: BMI, body mass index; EN, enteral nutrition; NPV, negative predictive value; PN, parenteral nutrition; PNST, Pediatric Nutrition Screening Tool; PPV, positive predictive value; RD, registered dietitian; WFL, weight-for-length.

**Table 6:** Comparison to the Pediatric Nutrition Screening Tool *.

Tool	Age Group	Malnutrition Definition	Sensitivity	Specificity	PPV	NPV
Children’s Wisconsin Nutrition Screening Tool	0–17 years	All levels of malnutrition (mild, moderate, and severe)	98.5%	6.0%	24.9%	92.9%
		Moderate or severe nutrition	100%	5.2%	5.2%	100%
Pediatric Nutrition Screening Tool in the present study		All levels of malnutrition (mild, moderate, and severe)	10.0%	98.1%	65.8%	74.9%
		Moderate or severe malnutrition	14.1%	97.5%	47.4%	87.8%
Pediatric Nutrition Screening Tool (original validation study) [9]	0–16 years	BMI z-score < −2	89.3%	66.2%	22.5%	98.4%
		BMI z-score < −3	100%	62.8%	5.4%	100%

Legend: NPV, negative predictive value; PPV, positive predictive value. Note: * The study methodology undoubtedly contributed to the results (i.e.,) these statistics are based upon children with a positive screen. The original PNST is for reference only as these data were derived using a different study methodology.

## Data Availability

Data are unavailable for privacy reasons.
